# Supplementary material for: Rubia akane Nakai Fruit Extract Improves Obesity and Insulin Sensitivity in 3T3-L1 Adipocytes and High-Fat Diet-Induced Obese Mice
Source: Int J Mol Sci. 2025 Feb 20;26(5):1833. doi: 10.3390/ijms26051833 (PMC11899715; doi:10.3390/ijms26051833)
Supplement: Supplementary file 1 [file ijms-26-01833-s001.zip › ijms-3440094-supplementary.pdf]

International Journal of *Molecular Sciences*

MDPI

Supplementary Information

Rubia akane Nakai Fruit Extract Improves Obesity and Insulin Sensitivity in 3T3-L1 Adipocytes and High-Fat Diet-Induced Obese Mice

Juhye Park [1,†], Eunbi Lee [1,†] and Ju-Ock Nam [1,2,*]

1 School of Food Science and Biotechnology, College of Agriculture and Life Sciences, Kyungpook National University, Dague 41566, Republic of Korea; pdw8609@knu.ac.kr (J.P.); 21eunbi@knu.ac.kr (E.L.)
2 Research Institute of Tailored Food Technology, Kyungpook National University, Dague 41566, Republic of Korea
* Correspondence: namjo@knu.ac.kr; Tel.: +82-053-950-7760
† These authors contributed equally to this work.

Table S1. Effects of RFE on the weight of other metabolic organs

	ND	HFD	RFE 12.5	RFE 25
**iBAT**	0.062 ± 0.0050	0.125 ± 0.0152	0.101 ± 0.0057	0.116 ± 0.0120
**Liver**	1.016 ± 0.0286	1.414 ± 0.1202	1.408 ± 0.1054	1.287 ± 0.1005
**Spleen**	0.064 ± 0.0017	0.095 ± 0.0076	0.093 ± 0.0044	0.095 ± 0.0041
**Kidney**	0.308 ± 0.0084	0.338 ± 0.0138	0.335 ± 0.0106	0.335 ± 0.0196
**GAS muscle**	0.319 ± 0.0154	0.356 ± 0.0224	0.348 ± 0.0106	0.359 ± 0.0177

Table S2. Effects of RFE on biochemical parameters of liver and kidney damage

	ND	HFD	RFE 12.5	RFE 25
**GOT (U/L)**	99.77 ± 23.397	146.95 ± 29.850	136.27 ± 16.775	128.95 ± 12.550
**GPT (U/L)**	45.00 ± 13.032	95.67 ± 25.693	92.33 ± 18.003	91.33 ± 6.888
**BUN (mg/dL)**	23.55 ± 2.950	21.17 ± 0.088	19.05 ± 0.999	18.80 ± 2.212
**CREA (mg/dL)**	0.47 ± 0.044	0.48 ± 0.012	0.46 ± 0.014	0.47 ± 0.018

Table S3. Primer sequences used for RT-qPCR

Gene name		Primer Sequence
Pparγ	Forward	5'-TTTTCAAGGGTGCCAGTTTC-3'
	Reverse	3'-AATCCTTGGCCCTCTGAGAT-5'
C/ebpα	Forward	5'-TTACAACAGGCCAGGTTTCC-3'
	Reverse	3'-GGCTGGCGACATACAGATCA-5'
Gata2	Forward	5'-GCAGAGAAGCAAGGCTCGC-3'
	Reverse	3'-CGGCCCTCACACAGTTGAC-5'
Chop10	Forward	5'-GTGGGTAGCTTGGCTGACA-3'
	Reverse	3'-GTTTCGGGAGCGAGAGGT-5'
Adipoq	Forward	5′-ACCTACGACCAGTATCAGGAAAAG-3′
	Reverse	3′-ACTAAGCTGAAAGTGTGTCGACTG-5′
Fas	Forward	5'-GGTCGTTTCTCCATTAAATTCTCAT-3'
	Reverse	3'-CTAGAAACTTTCCCAGAAATCTTCC-5'
Lpl	Forward	5'-CTGGTGGGAAATGATGTGG-3'
	Reverse	3'-TGGACGTTGTCTAGGGGGTA-5'
Ap2	Forward	5'-AAGGTGAAGAGCATAACCCT-3'

	Reverse	3'-TCACGCCTTTCATAACACATTCC-5'
β-actin	Forward	5'-GACAACGGCTCCGGCATGTGCAAAG-3'
	Reverse	3'-TTCACGGTTGGCCTTAGGGTTCAG-5'

Table S4. Primary antibodies used for Western blot analysis

Gene name	Company	Product No.	Dilution
PPARγ	Santa Cruz Biotechnology	sc-7273	1:500
C/EBPα	Cell Signaling Technology	2295S	1:1000
SREBP1	Cell Signaling Technology	sc-365513	1:500
AMPK	Cell Signaling Technology	2532S	1:1000
p-AMPK	Cell Signaling Technology	2531S	1:1000
GLUT4	Cell Signaling Technology	2213S	1:1000
β-actin	Santa Cruz Biotechnology	sc-47778	1:500

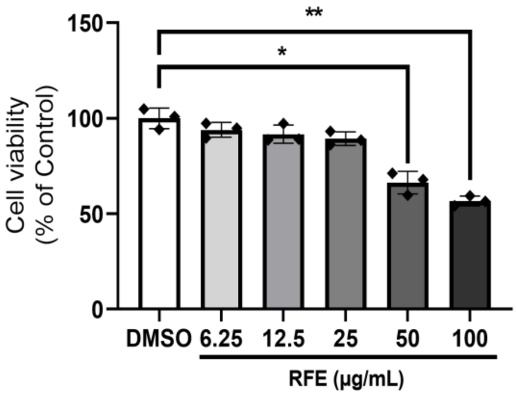

Figure S1. Effects of RFE on the cytotoxicity of 3T3-L1 preadipocytes. To evaluate the cytotoxicity of RFE on 3T3-L1 preadipocytes, cells were treated with various of doses (6.25–100 μg/mL) of RFE or DMSO (control; dimethyl sulfoxide) for 24 h, and the MTT assay was employed to ascertain cell viability. An extract was considered cytotoxic if the viability of 3T3-L1 preadipocytes fell below 80% compared to the control (DMSO). Three biologically and technically independent repetitions were undertaken for all experiments, and the results are depicted as the mean ± standard deviation (SD). * $p < 0.05$, ** $p < 0.01$ compared with the DMSO group.

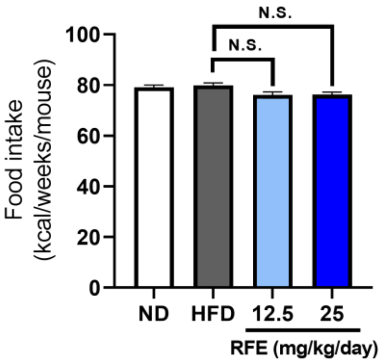

Figure S2. Effects of RFE on food intake. The graph shows the mean weekly caloric intake of mice in each group. Each mouse's food intake was recorded once a week for 12 weeks. Bar graphs represent the mean ± standard error of the mean (SEM). N.S. indicates no statistically significant difference compared with the HFD group (control).

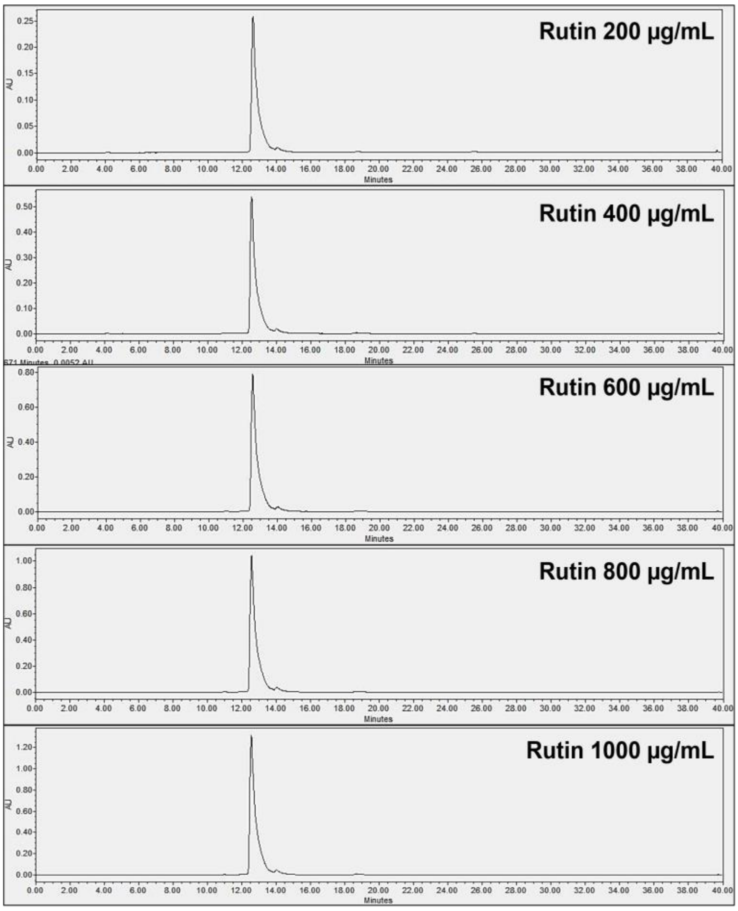

µg/mL	Area (µV*sec)
0	0
200	16625298
400	33669357
600	50382132
800	68738904
1000	87776220

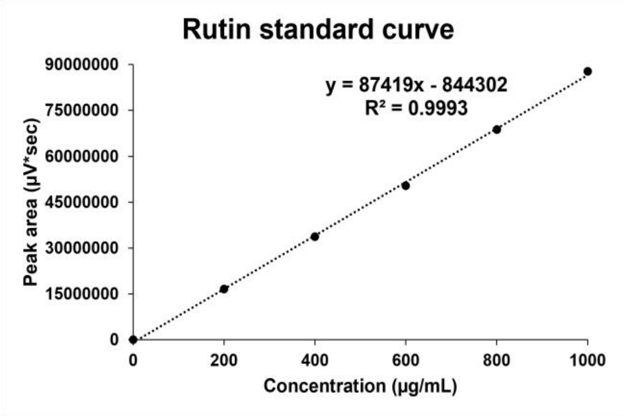

	Area (µV*sec)		Concentration
Rutin (in 30 µL RFE)	2001969	32.56 µg/30 µL	**1.085 mg/mL**

Figure S3. Quantification of rutin concentration included in RFE. To quantify rutin concentration in RFE, a standard curve of rutin was obtained by HPLC analysis. (X: concentration (µg/mL), Y: peak area (µV*sec)).
